# Bis(2-cyclo­hexyl­imino­methyl-4,6-dihydro­seleno­phenolato)cobalt(II) acetonitrile solvate

**DOI:** 10.1107/S1600536809034795

**Published:** 2009-09-09

**Authors:** Yong-Ming Cui, Xian Zhang, Li-Jun Wang, Qing-Fu Zeng

**Affiliations:** a Engineering Research Center for Clean Production of Textile Dyeing and Printing, Ministry of Education, Wuhan 430073, People’s Republic of China

## Abstract

In the title compound, [Co(C_13_H_16_NOSe_2_)_2_]·CH_3_CN, the Co^II^ atom is four-coordinated by two *N*,*O*-bidentate Schiff base ligands, resulting in a distorted tetra­hedral coordination for the metal ion.

## Related literature

For background to Schiff bases, see: Shi *et al.* (2007 ▶, 2008 ▶). For reference structural data, see: Allen *et al.* (1987 ▶).
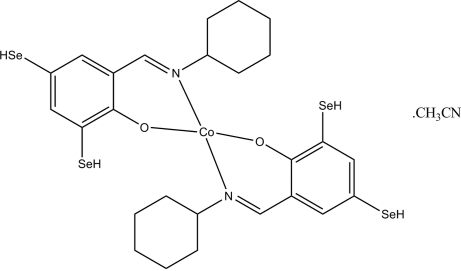


## Experimental

### 

#### Crystal data

[Co(C_13_H_16_NOSe_2_)_2_]·C_2_H_3_N
*M* *_r_* = 820.36
Monoclinic, 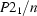

*a* = 9.4745 (5) Å
*b* = 16.3895 (5) Å
*c* = 20.4615 (5) Å
β = 91.845 (2)°
*V* = 3175.7 (2) Å^3^
*Z* = 4
Mo *K*α radiation
μ = 5.15 mm^−1^
*T* = 298 K
0.20 × 0.14 × 0.12 mm

#### Data collection

Enraf–Nonius CAD-4 diffractometer
Absorption correction: ψ scan (North *et al.*, 1968 ▶) *T*_min_ = 0.426, *T*_max_ = 0.577
30082 measured reflections
5592 independent reflections
4034 reflections with *I* > 2σ(*I*)
*R*_int_ = 0.043
200 standard reflections every 3 reflections intensity decay: 1%

#### Refinement

*R*[*F* ^2^ > 2σ(*F* ^2^)] = 0.044
*wR*(*F* ^2^) = 0.141
*S* = 1.08
5592 reflections
348 parameters
H-atom parameters constrained
Δρ_max_ = 0.88 e Å^−3^
Δρ_min_ = −0.83 e Å^−3^

### 

Data collection: *CAD-4 Software* (Enraf–Nonius, 1989 ▶); cell refinement: *CAD-4 Software*; data reduction: *XCAD4* (Harms & Wocadlo, 1995 ▶); program(s) used to solve structure: *SHELXS97* (Sheldrick, 2008 ▶); program(s) used to refine structure: *SHELXL97* (Sheldrick, 2008 ▶); molecular graphics: *SHELXTL* (Sheldrick, 2008 ▶); software used to prepare material for publication: *SHELXTL*.

## Supplementary Material

Crystal structure: contains datablocks global, I. DOI: 10.1107/S1600536809034795/hb5080sup1.cif

Structure factors: contains datablocks I. DOI: 10.1107/S1600536809034795/hb5080Isup2.hkl

Additional supplementary materials:  crystallographic information; 3D view; checkCIF report

## Figures and Tables

**Table 1:** Selected bond lengths (Å)

Co1—O1	1.904 (3)
Co1—O2	1.916 (3)
Co1—N1	1.999 (4)
Co1—N2	1.999 (4)

**Fig. 1.**
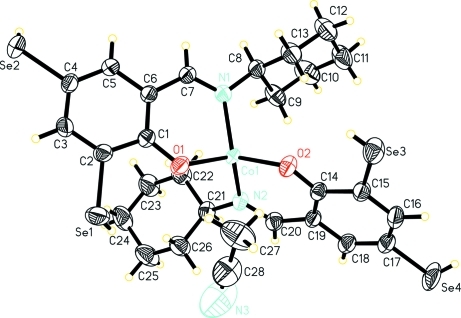
The structure of the title compound (I) showing 30% probability displacement ellipsoids.

## supplementary crystallographic information

### Comment

There has been much research interest in Schiff base metal complexes due to their molecular architectures and biological activities (Shi *et al.*, 2007; Shi *et al.*, 2008). In this work, we report here the crystal structure of the title compound, (I). In (I), all bond lengths are within normal ranges (Allen *et al.*, 1987) (Fig. 1). The Co^II^ is four-coordinated in a distorted tetrahedral coordination by two N atoms and two O atoms of the Schiff base ligand.

### Experimental

A mixture of 3,5-dihydroseleno-2-hydroxybenzaldehyde (564 mg, 2 mmol), cyclohexanamine (198 mg, 2 mmol) and CoCl_2_.6H_2_O (1 mmol, 238 mg) was stirred in acetonitrile (10 ml) for 1 h. After keeping the filtrate in air for 8 d, blue blocks of (I) were formed.

### Refinement

All H atoms were positioned geometrically (C—H = 0.93 Å for the aromatic H atoms and C—H = 0.96 Å for the aliphatic H atoms) and were refined as riding, with *U*_iso_(H) = 1.2*U*_eq_(C) and *U*_iso_(H) = 1.2*U*_eq_(N).

### Crystal data

**Table:**

[Co(C_13_H_16_NOSe_2_)_2_]·C_2_H_3_N	*F*(000) = 1612
*M**_r_* = 820.36	*D*_x_ = 1.716 Mg m^−^^3^
Monoclinic, *P*2_1_/*n*	Mo *K*α radiation, λ = 0.71073 Å
Hall symbol: -P 2yn	Cell parameters from 25 reflections
*a* = 9.4745 (5) Å	θ = 9–12°
*b* = 16.3895 (5) Å	µ = 5.15 mm^−^^1^
*c* = 20.4615 (5) Å	*T* = 298 K
β = 91.845 (2)°	Block, blue
*V* = 3175.7 (2) Å^3^	0.20 × 0.14 × 0.12 mm
*Z* = 4	

### Data collection

**Table:**

Enraf–Nonius CAD-4 diffractometer	4034 reflections with *I* > 2σ(*I*)
Radiation source: fine-focus sealed tube	*R*_int_ = 0.043
graphite	θ_max_ = 25.0°, θ_min_ = 1.6°
ω/2θ scans	*h* = −11→11
Absorption correction: ψ scan (North *et al.*, 1968)	*k* = −19→19
*T*_min_ = 0.426, *T*_max_ = 0.577	*l* = −23→24
30082 measured reflections	200 standard reflections every 3 reflections
5592 independent reflections	intensity decay: 1%

### Refinement

**Table:**

Refinement on *F*^2^	Primary atom site location: structure-invariant direct methods
Least-squares matrix: full	Secondary atom site location: difference Fourier map
*R*[*F*^2^ > 2σ(*F*^2^)] = 0.044	Hydrogen site location: inferred from neighbouring sites
*wR*(*F*^2^) = 0.141	H-atom parameters constrained
*S* = 1.08	*w* = 1/[σ^2^(*F*_o_^2^) + (0.0729*P*)^2^ + 2.8479*P*] where *P* = (*F*_o_^2^ + 2*F*_c_^2^)/3
5592 reflections	(Δ/σ)_max_ < 0.001
348 parameters	Δρ_max_ = 0.88 e Å^−^^3^
0 restraints	Δρ_min_ = −0.83 e Å^−^^3^

### Special details

**Table:**

Geometry. All e.s.d.'s (except the e.s.d. in the dihedral angle between two l.s. planes) are estimated using the full covariance matrix. The cell e.s.d.'s are taken into account individually in the estimation of e.s.d.'s in distances, angles and torsion angles; correlations between e.s.d.'s in cell parameters are only used when they are defined by crystal symmetry. An approximate (isotropic) treatment of cell e.s.d.'s is used for estimating e.s.d.'s involving l.s. planes.
Refinement. Refinement of *F*^2^ against ALL reflections. The weighted *R*-factor *wR* and goodness of fit *S* are based on *F*^2^, conventional *R*-factors *R* are based on *F*, with *F* set to zero for negative *F*^2^. The threshold expression of *F*^2^ > σ(*F*^2^) is used only for calculating *R*-factors(gt) *etc*. and is not relevant to the choice of reflections for refinement. *R*-factors based on *F*^2^ are statistically about twice as large as those based on *F*, and *R*- factors based on ALL data will be even larger.

### Fractional atomic coordinates and isotropic or equivalent isotropic displacement parameters (Å^2^)

**Table:**

	*x*	*y*	*z*	*U*_iso_*/*U*_eq_	
Co1	0.71461 (6)	0.25063 (3)	0.18676 (3)	0.03902 (18)	
Se1	0.78872 (10)	0.32963 (5)	0.40908 (3)	0.0967 (3)	
H1	0.7785	0.3664	0.3821	0.145*	
Se2	1.23778 (8)	0.10610 (5)	0.43164 (3)	0.0848 (2)	
H2	1.3033	0.0961	0.4077	0.127*	
Se3	0.26742 (7)	0.13073 (4)	0.12309 (4)	0.0831 (2)	
H3A	0.3336	0.1105	0.1442	0.125*	
Se4	0.14412 (10)	0.40993 (5)	−0.02853 (5)	0.1130 (3)	
H4	0.1026	0.3769	−0.0523	0.169*	
C1	0.8578 (5)	0.2201 (3)	0.3084 (2)	0.0512 (12)	
C2	0.8960 (6)	0.2438 (3)	0.3727 (3)	0.0576 (13)	
C3	1.0044 (6)	0.2114 (3)	0.4092 (3)	0.0630 (15)	
H3	1.0245	0.2293	0.4516	0.076*	
C4	1.0837 (6)	0.1511 (3)	0.3810 (3)	0.0568 (13)	
C5	1.0558 (6)	0.1246 (3)	0.3186 (3)	0.0561 (13)	
H5	1.1106	0.0835	0.3010	0.067*	
C6	0.9441 (5)	0.1593 (3)	0.2806 (2)	0.0493 (12)	
C7	0.9286 (5)	0.1301 (3)	0.2144 (2)	0.0520 (12)	
H7	0.9847	0.0859	0.2034	0.062*	
C8	0.8488 (6)	0.1210 (3)	0.1028 (2)	0.0590 (13)	
H8	0.9382	0.0917	0.0987	0.071*	
C9	0.8394 (8)	0.1865 (4)	0.0513 (3)	0.0756 (18)	
H9A	0.9183	0.2237	0.0574	0.091*	
H9B	0.7530	0.2174	0.0562	0.091*	
C10	0.8410 (10)	0.1508 (5)	−0.0167 (3)	0.101 (3)	
H10A	0.9304	0.1236	−0.0229	0.121*	
H10B	0.8318	0.1944	−0.0487	0.121*	
C11	0.7212 (10)	0.0904 (4)	−0.0274 (3)	0.106 (3)	
H11A	0.7275	0.0658	−0.0703	0.127*	
H11B	0.6316	0.1188	−0.0258	0.127*	
C12	0.7271 (10)	0.0240 (4)	0.0245 (3)	0.096 (2)	
H12A	0.8114	−0.0087	0.0195	0.115*	
H12B	0.6457	−0.0115	0.0188	0.115*	
C13	0.7288 (7)	0.0613 (4)	0.0934 (3)	0.0749 (17)	
H13A	0.6399	0.0890	0.1001	0.090*	
H13B	0.7385	0.0181	0.1257	0.090*	
C14	0.4488 (5)	0.2682 (3)	0.1182 (2)	0.0471 (11)	
C15	0.3182 (5)	0.2362 (3)	0.0947 (3)	0.0576 (13)	
C16	0.2303 (6)	0.2780 (4)	0.0515 (3)	0.0655 (15)	
H16	0.1455	0.2548	0.0368	0.079*	
C17	0.2684 (6)	0.3539 (4)	0.0304 (3)	0.0653 (15)	
C18	0.3922 (6)	0.3876 (4)	0.0509 (3)	0.0645 (14)	
H18	0.4162	0.4393	0.0362	0.077*	
C19	0.4854 (5)	0.3461 (3)	0.0941 (2)	0.0514 (12)	
C20	0.6163 (6)	0.3879 (3)	0.1118 (3)	0.0573 (13)	
H20	0.6259	0.4402	0.0949	0.069*	
C21	0.8396 (6)	0.4181 (3)	0.1603 (3)	0.0614 (14)	
H21	0.8476	0.4544	0.1225	0.074*	
C22	0.9765 (6)	0.3710 (4)	0.1690 (4)	0.0810 (19)	
H22A	0.9935	0.3400	0.1296	0.097*	
H22B	0.9689	0.3329	0.2049	0.097*	
C23	1.1006 (7)	0.4290 (5)	0.1830 (4)	0.096 (2)	
H23A	1.1863	0.3975	0.1904	0.115*	
H23B	1.1137	0.4638	0.1453	0.115*	
C24	1.0754 (7)	0.4805 (5)	0.2413 (4)	0.096 (2)	
H24A	1.1532	0.5184	0.2478	0.116*	
H24B	1.0715	0.4461	0.2798	0.116*	
C25	0.9388 (8)	0.5275 (5)	0.2329 (4)	0.102 (2)	
H25A	0.9458	0.5654	0.1968	0.123*	
H25B	0.9225	0.5587	0.2723	0.123*	
C26	0.8150 (7)	0.4698 (4)	0.2196 (4)	0.084 (2)	
H26A	0.8030	0.4348	0.2573	0.101*	
H26B	0.7291	0.5013	0.2128	0.101*	
C27	0.3987 (11)	0.2810 (6)	0.3050 (5)	0.129 (3)	
H27A	0.4115	0.2561	0.2631	0.193*	
H27B	0.3041	0.2714	0.3185	0.193*	
H27C	0.4645	0.2580	0.3365	0.193*	
C28	0.4218 (9)	0.3646 (6)	0.3003 (5)	0.114 (3)	
N1	0.8459 (4)	0.1584 (3)	0.1690 (2)	0.0520 (10)	
N2	0.7197 (4)	0.3621 (2)	0.14735 (19)	0.0513 (10)	
N3	0.4406 (11)	0.4297 (7)	0.2980 (7)	0.198 (6)	
O1	0.7504 (4)	0.2540 (2)	0.27880 (17)	0.0631 (10)	
O2	0.5246 (4)	0.2252 (2)	0.15900 (18)	0.0597 (9)	

### Atomic displacement parameters (Å^2^)

**Table:**

	*U*^11^	*U*^22^	*U*^33^	*U*^12^	*U*^13^	*U*^23^
Co1	0.0356 (3)	0.0429 (3)	0.0382 (3)	0.0049 (3)	−0.0047 (2)	0.0054 (3)
Se1	0.1520 (7)	0.0796 (5)	0.0579 (4)	0.0525 (5)	−0.0053 (4)	−0.0112 (3)
Se2	0.0823 (5)	0.0871 (5)	0.0825 (5)	0.0177 (4)	−0.0361 (4)	0.0025 (4)
Se3	0.0677 (4)	0.0769 (4)	0.1036 (5)	−0.0214 (3)	−0.0137 (4)	0.0174 (4)
Se4	0.0972 (6)	0.1095 (6)	0.1279 (7)	0.0072 (5)	−0.0632 (5)	0.0329 (5)
C1	0.054 (3)	0.051 (3)	0.048 (3)	0.003 (2)	−0.004 (2)	0.005 (2)
C2	0.074 (4)	0.052 (3)	0.047 (3)	0.012 (3)	0.001 (3)	0.003 (2)
C3	0.087 (4)	0.051 (3)	0.050 (3)	0.001 (3)	−0.018 (3)	0.000 (2)
C4	0.056 (3)	0.053 (3)	0.059 (3)	0.000 (2)	−0.015 (3)	0.007 (2)
C5	0.055 (3)	0.050 (3)	0.063 (3)	0.006 (2)	−0.008 (3)	−0.003 (2)
C6	0.048 (3)	0.043 (3)	0.056 (3)	−0.001 (2)	−0.007 (2)	0.001 (2)
C7	0.048 (3)	0.056 (3)	0.052 (3)	0.007 (2)	−0.002 (2)	−0.003 (2)
C8	0.059 (3)	0.069 (3)	0.049 (3)	0.008 (3)	−0.003 (2)	−0.009 (3)
C9	0.106 (5)	0.069 (4)	0.053 (3)	−0.026 (3)	0.013 (3)	−0.005 (3)
C10	0.162 (8)	0.086 (5)	0.056 (4)	−0.018 (5)	0.023 (4)	0.004 (3)
C11	0.174 (8)	0.082 (5)	0.058 (4)	−0.013 (5)	−0.040 (4)	0.003 (3)
C12	0.151 (7)	0.067 (4)	0.068 (4)	−0.019 (4)	−0.015 (4)	−0.005 (3)
C13	0.109 (5)	0.057 (3)	0.058 (3)	−0.011 (3)	−0.009 (3)	0.008 (3)
C14	0.041 (3)	0.058 (3)	0.042 (3)	0.003 (2)	0.002 (2)	0.003 (2)
C15	0.048 (3)	0.068 (3)	0.056 (3)	0.004 (2)	−0.003 (2)	−0.003 (3)
C16	0.047 (3)	0.087 (4)	0.062 (3)	−0.003 (3)	−0.009 (3)	−0.005 (3)
C17	0.061 (4)	0.067 (4)	0.067 (4)	0.009 (3)	−0.016 (3)	0.008 (3)
C18	0.065 (4)	0.063 (3)	0.065 (3)	0.006 (3)	−0.011 (3)	0.008 (3)
C19	0.047 (3)	0.053 (3)	0.053 (3)	0.003 (2)	−0.008 (2)	0.004 (2)
C20	0.063 (3)	0.054 (3)	0.054 (3)	−0.001 (3)	−0.004 (3)	0.008 (2)
C21	0.062 (3)	0.057 (3)	0.064 (3)	−0.011 (3)	−0.009 (3)	0.011 (3)
C22	0.049 (3)	0.088 (4)	0.106 (5)	−0.009 (3)	0.004 (3)	−0.027 (4)
C23	0.060 (4)	0.106 (5)	0.121 (6)	−0.015 (4)	0.001 (4)	−0.031 (5)
C24	0.075 (5)	0.113 (6)	0.100 (5)	−0.030 (4)	0.000 (4)	−0.034 (5)
C25	0.087 (5)	0.092 (5)	0.130 (6)	−0.020 (4)	0.012 (5)	−0.046 (5)
C26	0.066 (4)	0.082 (4)	0.103 (5)	−0.005 (3)	0.002 (4)	−0.034 (4)
C27	0.153 (9)	0.122 (7)	0.115 (7)	−0.018 (6)	0.055 (6)	−0.023 (6)
C28	0.092 (6)	0.104 (7)	0.146 (8)	−0.022 (5)	0.003 (5)	0.023 (6)
N1	0.051 (2)	0.055 (2)	0.050 (2)	0.0023 (19)	−0.001 (2)	−0.0058 (19)
N2	0.047 (2)	0.055 (2)	0.052 (2)	−0.0031 (19)	−0.005 (2)	0.0063 (19)
N3	0.138 (8)	0.126 (7)	0.326 (17)	−0.033 (6)	−0.034 (9)	0.068 (9)
O1	0.066 (2)	0.075 (2)	0.048 (2)	0.0262 (19)	−0.0044 (18)	−0.0012 (17)
O2	0.051 (2)	0.062 (2)	0.066 (2)	−0.0037 (17)	−0.0102 (18)	0.0153 (18)

### Geometric parameters (Å, °)

**Table:**

Co1—O1	1.904 (3)	C12—H12B	0.9700
Co1—O2	1.916 (3)	C13—H13A	0.9700
Co1—N1	1.999 (4)	C13—H13B	0.9700
Co1—N2	1.999 (4)	C14—O2	1.293 (6)
Se1—C2	1.901 (5)	C14—C15	1.414 (7)
Se1—H1	0.8200	C14—C19	1.415 (7)
Se2—C4	1.910 (5)	C15—C16	1.377 (7)
Se2—H2	0.8200	C16—C17	1.368 (8)
Se3—C15	1.890 (6)	C16—H16	0.9300
Se3—H3A	0.8200	C17—C18	1.351 (8)
Se4—C17	1.896 (5)	C18—C19	1.405 (7)
Se4—H4	0.8200	C18—H18	0.9300
C1—O1	1.293 (6)	C19—C20	1.452 (7)
C1—C2	1.408 (7)	C20—N2	1.273 (6)
C1—C6	1.419 (7)	C20—H20	0.9300
C2—C3	1.359 (7)	C21—N2	1.477 (6)
C3—C4	1.379 (8)	C21—C26	1.504 (8)
C3—H3	0.9300	C21—C22	1.516 (8)
C4—C5	1.367 (7)	C21—H21	0.9800
C5—C6	1.413 (7)	C22—C23	1.532 (8)
C5—H5	0.9300	C22—H22A	0.9700
C6—C7	1.439 (7)	C22—H22B	0.9700
C7—N1	1.283 (6)	C23—C24	1.486 (10)
C7—H7	0.9300	C23—H23A	0.9700
C8—N1	1.487 (6)	C23—H23B	0.9700
C8—C9	1.505 (8)	C24—C25	1.511 (10)
C8—C13	1.508 (8)	C24—H24A	0.9700
C8—H8	0.9800	C24—H24B	0.9700
C9—C10	1.511 (9)	C25—C26	1.524 (9)
C9—H9A	0.9700	C25—H25A	0.9700
C9—H9B	0.9700	C25—H25B	0.9700
C10—C11	1.517 (10)	C26—H26A	0.9700
C10—H10A	0.9700	C26—H26B	0.9700
C10—H10B	0.9700	C27—C28	1.391 (13)
C11—C12	1.520 (9)	C27—H27A	0.9600
C11—H11A	0.9700	C27—H27B	0.9600
C11—H11B	0.9700	C27—H27C	0.9600
C12—C13	1.536 (9)	C28—N3	1.082 (11)
C12—H12A	0.9700		
			
O1—Co1—O2	115.82 (16)	C16—C15—C14	122.5 (5)
O1—Co1—N1	96.32 (16)	C16—C15—Se3	119.8 (4)
O2—Co1—N1	111.45 (16)	C14—C15—Se3	117.6 (4)
O1—Co1—N2	111.50 (16)	C17—C16—C15	119.6 (5)
O2—Co1—N2	96.55 (15)	C17—C16—H16	120.2
N1—Co1—N2	126.51 (17)	C15—C16—H16	120.2
C2—Se1—H1	109.5	C18—C17—C16	120.6 (5)
C4—Se2—H2	109.5	C18—C17—Se4	120.9 (4)
C15—Se3—H3A	109.5	C16—C17—Se4	118.4 (4)
C17—Se4—H4	109.5	C17—C18—C19	121.2 (5)
O1—C1—C2	119.6 (5)	C17—C18—H18	119.4
O1—C1—C6	124.7 (4)	C19—C18—H18	119.4
C2—C1—C6	115.8 (4)	C18—C19—C14	120.0 (5)
C3—C2—C1	124.9 (5)	C18—C19—C20	116.2 (5)
C3—C2—Se1	118.6 (4)	C14—C19—C20	123.8 (4)
C1—C2—Se1	116.5 (4)	N2—C20—C19	128.4 (5)
C2—C3—C4	117.6 (5)	N2—C20—H20	115.8
C2—C3—H3	121.2	C19—C20—H20	115.8
C4—C3—H3	121.2	N2—C21—C26	110.9 (5)
C5—C4—C3	121.7 (5)	N2—C21—C22	110.8 (5)
C5—C4—Se2	120.4 (4)	C26—C21—C22	110.2 (5)
C3—C4—Se2	117.9 (4)	N2—C21—H21	108.3
C4—C5—C6	120.4 (5)	C26—C21—H21	108.3
C4—C5—H5	119.8	C22—C21—H21	108.3
C6—C5—H5	119.8	C21—C22—C23	110.8 (6)
C5—C6—C1	119.5 (5)	C21—C22—H22A	109.5
C5—C6—C7	115.8 (4)	C23—C22—H22A	109.5
C1—C6—C7	124.6 (4)	C21—C22—H22B	109.5
N1—C7—C6	127.1 (5)	C23—C22—H22B	109.5
N1—C7—H7	116.5	H22A—C22—H22B	108.1
C6—C7—H7	116.5	C24—C23—C22	111.1 (6)
N1—C8—C9	110.0 (4)	C24—C23—H23A	109.4
N1—C8—C13	110.3 (5)	C22—C23—H23A	109.4
C9—C8—C13	110.3 (5)	C24—C23—H23B	109.4
N1—C8—H8	108.7	C22—C23—H23B	109.4
C9—C8—H8	108.7	H23A—C23—H23B	108.0
C13—C8—H8	108.7	C23—C24—C25	111.0 (6)
C8—C9—C10	111.5 (5)	C23—C24—H24A	109.4
C8—C9—H9A	109.3	C25—C24—H24A	109.4
C10—C9—H9A	109.3	C23—C24—H24B	109.4
C8—C9—H9B	109.3	C25—C24—H24B	109.4
C10—C9—H9B	109.3	H24A—C24—H24B	108.0
H9A—C9—H9B	108.0	C24—C25—C26	110.8 (6)
C9—C10—C11	110.8 (6)	C24—C25—H25A	109.5
C9—C10—H10A	109.5	C26—C25—H25A	109.5
C11—C10—H10A	109.5	C24—C25—H25B	109.5
C9—C10—H10B	109.5	C26—C25—H25B	109.5
C11—C10—H10B	109.5	H25A—C25—H25B	108.1
H10A—C10—H10B	108.1	C21—C26—C25	110.8 (6)
C10—C11—C12	110.9 (6)	C21—C26—H26A	109.5
C10—C11—H11A	109.5	C25—C26—H26A	109.5
C12—C11—H11A	109.5	C21—C26—H26B	109.5
C10—C11—H11B	109.5	C25—C26—H26B	109.5
C12—C11—H11B	109.5	H26A—C26—H26B	108.1
H11A—C11—H11B	108.1	C28—C27—H27A	109.5
C11—C12—C13	110.8 (6)	C28—C27—H27B	109.5
C11—C12—H12A	109.5	H27A—C27—H27B	109.5
C13—C12—H12A	109.5	C28—C27—H27C	109.5
C11—C12—H12B	109.5	H27A—C27—H27C	109.5
C13—C12—H12B	109.5	H27B—C27—H27C	109.5
H12A—C12—H12B	108.1	N3—C28—C27	178.6 (14)
C8—C13—C12	111.1 (5)	C7—N1—C8	118.9 (4)
C8—C13—H13A	109.4	C7—N1—Co1	120.7 (3)
C12—C13—H13A	109.4	C8—N1—Co1	120.5 (3)
C8—C13—H13B	109.4	C20—N2—C21	118.0 (4)
C12—C13—H13B	109.4	C20—N2—Co1	120.3 (4)
H13A—C13—H13B	108.0	C21—N2—Co1	121.6 (3)
O2—C14—C15	118.6 (4)	C1—O1—Co1	124.3 (3)
O2—C14—C19	125.4 (4)	C14—O2—Co1	124.7 (3)
C15—C14—C19	116.0 (4)		
			
O1—C1—C2—C3	178.5 (5)	C14—C19—C20—N2	3.8 (9)
C6—C1—C2—C3	−2.0 (8)	N2—C21—C22—C23	−179.5 (5)
O1—C1—C2—Se1	−3.1 (7)	C26—C21—C22—C23	−56.4 (8)
C6—C1—C2—Se1	176.4 (4)	C21—C22—C23—C24	56.5 (9)
C1—C2—C3—C4	0.4 (9)	C22—C23—C24—C25	−56.3 (9)
Se1—C2—C3—C4	−177.9 (4)	C23—C24—C25—C26	56.7 (9)
C2—C3—C4—C5	0.4 (8)	N2—C21—C26—C25	179.9 (6)
C2—C3—C4—Se2	179.3 (4)	C22—C21—C26—C25	56.9 (8)
C3—C4—C5—C6	0.5 (8)	C24—C25—C26—C21	−57.0 (9)
Se2—C4—C5—C6	−178.4 (4)	C6—C7—N1—C8	178.1 (5)
C4—C5—C6—C1	−2.1 (8)	C6—C7—N1—Co1	−2.2 (7)
C4—C5—C6—C7	176.8 (5)	C9—C8—N1—C7	−139.0 (5)
O1—C1—C6—C5	−177.8 (5)	C13—C8—N1—C7	99.1 (6)
C2—C1—C6—C5	2.7 (7)	C9—C8—N1—Co1	41.3 (6)
O1—C1—C6—C7	3.4 (8)	C13—C8—N1—Co1	−80.6 (5)
C2—C1—C6—C7	−176.1 (5)	O1—Co1—N1—C7	−7.3 (4)
C5—C6—C7—N1	−171.9 (5)	O2—Co1—N1—C7	−128.3 (4)
C1—C6—C7—N1	7.0 (8)	N2—Co1—N1—C7	115.4 (4)
N1—C8—C9—C10	−179.6 (6)	O1—Co1—N1—C8	172.4 (4)
C13—C8—C9—C10	−57.7 (7)	O2—Co1—N1—C8	51.4 (4)
C8—C9—C10—C11	57.4 (9)	N2—Co1—N1—C8	−64.9 (4)
C9—C10—C11—C12	−55.7 (9)	C19—C20—N2—C21	−177.5 (5)
C10—C11—C12—C13	54.8 (10)	C19—C20—N2—Co1	0.2 (8)
N1—C8—C13—C12	178.2 (5)	C26—C21—N2—C20	88.0 (6)
C9—C8—C13—C12	56.5 (7)	C22—C21—N2—C20	−149.2 (5)
C11—C12—C13—C8	−55.6 (9)	C26—C21—N2—Co1	−89.7 (5)
O2—C14—C15—C16	179.5 (5)	C22—C21—N2—Co1	33.0 (6)
C19—C14—C15—C16	−0.6 (8)	O1—Co1—N2—C20	−126.7 (4)
O2—C14—C15—Se3	−1.9 (6)	O2—Co1—N2—C20	−5.6 (4)
C19—C14—C15—Se3	177.9 (4)	N1—Co1—N2—C20	117.3 (4)
C14—C15—C16—C17	−0.6 (9)	O1—Co1—N2—C21	51.0 (4)
Se3—C15—C16—C17	−179.2 (4)	O2—Co1—N2—C21	172.0 (4)
C15—C16—C17—C18	0.9 (9)	N1—Co1—N2—C21	−65.1 (4)
C15—C16—C17—Se4	−179.6 (4)	C2—C1—O1—Co1	162.9 (4)
C16—C17—C18—C19	0.1 (9)	C6—C1—O1—Co1	−16.6 (7)
Se4—C17—C18—C19	−179.4 (4)	O2—Co1—O1—C1	134.1 (4)
C17—C18—C19—C14	−1.4 (8)	N1—Co1—O1—C1	16.5 (4)
C17—C18—C19—C20	178.9 (5)	N2—Co1—O1—C1	−116.9 (4)
O2—C14—C19—C18	−178.6 (5)	C15—C14—O2—Co1	170.8 (4)
C15—C14—C19—C18	1.6 (7)	C19—C14—O2—Co1	−9.0 (7)
O2—C14—C19—C20	1.0 (8)	O1—Co1—O2—C14	127.7 (4)
C15—C14—C19—C20	−178.8 (5)	N1—Co1—O2—C14	−123.5 (4)
C18—C19—C20—N2	−176.6 (6)	N2—Co1—O2—C14	10.0 (4)
